# 

**Published:** 1878-05

**Authors:** 


### Contents of May Number.

### ORIGINAL.

ART. I.—Median Lithotomy. By Charles A. Wall, B. S.,.... ....... 361
ART. II.—Recto-Vaginal Fistula. Operation by Prof. J. F. Miner, M. D. Reported by Charles G. Stockton, M. D., Resident Physician,........ 370
ART. III.—Extract of Malt and Phosphorus Compounds in the Treatment of Phthisis. By E. C. Lester, M. D.,........ 373
ART. IV.—Abstract of the Proceedings of the Buffalo Medical Association. Reported by Joseph Fowler, M. D., Sec.,........ 376

### MISCELLANEOUS.

Medical Experts as Witnesses in Courts of Justice, 378. Inverted Uterus of Forty Years Standing, Reduced in Half an Hour,—A new method, 381. Eucalyptus Tree, 383. Dr. W. A. Hammond, 383. The General Hospital in Vienna, 384. Mercurial Treatment of Syphilis, 384. Contra-Indications of Iron, 385. The External Use of Tincture of Belladonna in Night-Sweating, 385. Treatment of Tetanus, 386. Treatment of Delirium Tremens, 386. Arsenic Poisoning Successfully Treated by Dialysed Iron, 387. A Medical Opinion, 387. Ovariotomy at the Samaritan Hospital, 388. The Easiest Way to Drown, 388. Death of the Discoverer of Fœtal Auscultation, 388. Treatment of Epilepsy, 389. Carbonate of Soda, in Whooping Cough, 389. Threatened Poisoning of the Rhine, 390.

### EDITORIAL.

American Medical Association,........ 390
Accomodations for Delegates,........ 390
Circular of the Permanent Secretary,........ 390
Notice by the Committee of Arrangements,........ 393
American Medical College Association,........ 394
Medical Notes and Comments. By Chas. A. Wall, B. S.,........ 394
Notes on Exchanges,........ 395–396

BOOK NOTICES :

Atlas of Skin Diseases. Duhring,........ 396
State Regulation of Vice. Powell,........ 397
Mortuary Experience of the Mutual Life Ins. Co.,........ 397
Transactions of the Ohio State Medical Association,........ 397
The Multum in Parvo Reference and Dose Book. Leonard,... 397

BOOKS REVIEWED:

Hand Book of the Practice of Medicine. Charteris,........ 398
Transactions of the American Medical Association,........ 398
The Science and Art of Surgery. Erichsen,........ 399
Address before the Rocky Mountain Med. Ass. Goner,........ 398
Our Advertisers,........ 399

Books and Pamphlets Received,........ 399

THE BUFFALO

